# Toward an Efficient Method of Identifying Core Genes for Evolutionary and Functional Microbial Phylogenies

**DOI:** 10.1371/journal.pone.0024704

**Published:** 2011-09-12

**Authors:** Nicola Segata, Curtis Huttenhower

**Affiliations:** Biostatistics Department, Harvard School of Public Health, Harvard University, Boston, Massachusetts, United States of America; University of North Carolina at Charlotte, United States of America

## Abstract

Microbial community metagenomes and individual microbial genomes are becoming increasingly accessible by means of high-throughput sequencing. Assessing organismal membership within a community is typically performed using one or a few taxonomic marker genes such as the 16S rDNA, and these same genes are also employed to reconstruct molecular phylogenies. There is thus a growing need to bioinformatically catalog strongly conserved core genes that can serve as effective taxonomic markers, to assess the agreement among phylogenies generated from different core gene, and to characterize the biological functions enriched within core genes and thus conserved throughout large microbial clades. We present a method to recursively identify core genes (i.e. genes ubiquitous within a microbial clade) in high-throughput from a large number of complete input genomes. We analyzed over 1,100 genomes to produce core gene sets spanning 2,861 bacterial and archaeal clades, ranging in size from one to >2,000 genes in inverse correlation with the α-diversity (total phylogenetic branch length) spanned by each clade. These cores are enriched as expected for housekeeping functions including translation, transcription, and replication, in addition to significant representations of regulatory, chaperone, and conserved uncharacterized proteins. In agreement with previous manually curated core gene sets, phylogenies constructed from one or more of these core genes agree with those built using 16S rDNA sequence similarity, suggesting that systematic core gene selection can be used to optimize both comparative genomics and determination of microbial community structure. Finally, we examine functional phylogenies constructed by clustering genomes by the presence or absence of orthologous gene families and show that they provide an informative complement to standard sequence-based molecular phylogenies.

## Introduction

The number of fully sequenced microbial genomes recently passed one thousand, and the number of metagenomically sequenced microbial communities is also growing rapidly [1], [2]. Gene families strongly conserved within groups of related microbial organisms serve several important purposes in biologically interpreting these data. Their sequence variation can be used to reconstruct molecular phylogenies describing the evolutionary relationship among microorganisms [3], [4]. Further, the biological roles and molecular functions encoded by such conserved genes can provide insights into the phenotypes selected for within their containing microbial clades [5]. Finally, the variable regions of gene sequences shared by broad groups of bacteria or archaea can be used as taxonomic markers to determine their presence and abundance within microbial communities [6]. Each of these applications has been highly successful when employing manually curated core gene sets [3], [7], but as detailed below, current computational techniques rarely scale to thousands of complete genomes. Methods for rapidly cataloging gene families conserved within microbial clades are thus needed in order to take advantage of this growing number of sequenced organisms and communities.

Here, we use the common definition of “core genes” to refer to one or more gene families strongly conserved at the nucleotide sequence level throughout a related group of genomes [8]. The majority of existing approaches for identifying and leveraging core genes rely on sequence clustering algorithms, molecular phylogenetics, and gene functional annotation. The first approach, sequence clustering, identifies genes that are characteristic (i.e. present in almost all organisms) of a clade (note that this is distinct from the definition of “core genes” as referring to the minimal set of genes needed to sustain microbial life [9]). Core genes have been crucial in biologically investigating specific broad microbial clades including the *Cyanobacteria* [10], *Archaea* [11] and *Gammaproteobacteria* [12], [13] as well as viral clades [14] and specific species such as *Escherichia coli* [15]. Computational methods for systematically identifying core genes within clades spanning the entire bacteria and archaea have been proposed by [16] - 45 genomes, [17] - at most 5 genomes, [8] - 147 genomes, [18] - 175 genomes, and [19] - 205 proteomes. All of these examples focus only on genes shared by all bacteria exploiting local protein similarities (i.e. matching of fractions of the gene sequences) under the assumption that even a partial matching of a protein domain is indicative of functional relatedness. These existing systems can thus successfully identify a functional core at high phylogenetic levels, but this may come at the cost of neglecting more specific cores among more related clades. When more specific taxonomic units need to be investigated, however, strong conservation of the nucleotide sequence is crucial to identify shared genes; this is the case, for example, among *Escherichia coli* [15] as well as other clades [20], [21], [22]. In addition to this biological drawback, these existing approaches are also computationally infeasible to scale to the >1,200 fully annotated microbial genomes currently available.

Once defined, core gene sequences can of course be used to reconstruct molecular phylogenies, and this has typically been performed using a more general definition of core genes in which a core must be shared by a large fraction of genomes (but not necessarily by all of them). The common approach of several successful methods [3], [4], [16], [23], [24] consists of aligning and concatenating the orthologs of core genes in the genomes (allowing for missing orthologs) and building a phylogenetic tree using the maximum likelihood principle [25] or similar techniques. However, the most popular microbial “core gene” by far has been the single 16S rRNA gene [6], which fits the criteria of ubiquity, regions of strong conservation, and regions of hypervariability. It is supported by large datasets of sequenced orthologs [26], and essentially no single genes apart from 16S rDNA and ribosomal genes have been analyzed as single sources of phylogenetic information.

Once core genes have been defined to identify related groups of microbes, the biological roles performed by these genes' products can provide insights into the functions and phenotypes that may also be characteristic of the groups. Alternatively, rather than grouping organisms by molecular phylogeny and then determining what functions are conserved, it is possible to construct a functional phylogeny directly by grouping together organisms that contain similar complements of protein functional categories (using, for example, the COG system [27]). Such a functional phylogeny will cluster organisms with similar pathway representation and metabolic potential, regardless of their evolutionary relations, providing a complementary perspective on organisms that might share phenotypes or niches but not direct evolutionary ancestry. A similar approach is used in phylogenetic profiling to identify functional modules of related genes or proteins co-conserved across evolutionary time [28], [29], [30], [31]. Some initial work has constructed functional phylogenies for 55 genomes [32] and for 66 genomes [33], but it is as yet unclear how such information complements sequence-based phylogeny, scales to thousands of genomes, informs the functional analysis of microbial communities, or how it can be leveraged to compare organismal rather than biomolecular relationships.

In this work, we propose a computational method for core gene discovery and analysis in these three complementary areas. We define core genes hierarchically by searching for orthologs within a user-defined input taxonomy, thus avoiding computational issues that arise in applying brute force approaches to thousands of genomes. Our method produces over 2,000 core gene sets from the ∼1,100 available sequenced genomes within a few hours, identifying multiple core genes for clades ranging from the phylum to the strain levels and varying in size, as expected, proportionally to the phylogenetic diversity of each clade. We functionally characterized these core gene families to determine the protein functions evolutionarily conserved within each clade, which included both the expected housekeeping genes and a striking number of uncharacterized proteins. Molecular phylogenetic trees built using the sequences of core genes compare favorably with the common single 16S gene approach. In many cases, functional phylogenies constructed using the co-occurrence of protein families also agreed with these molecular phylogenies, thus quantifying a portion of the link between genome sequence and organismal functional potential. This computational framework thus allows the relatively short sequences of core gene families to serve both as markers for microbial phylogeny and as functional indicators for the phenotypes of microbial organisms and communities.

## Methods

### Core gene discovery algorithm

We applied our method for core gene discovery to the set of 1,236 prokaryotic genomes currently available from the NCBI (December 2010). Using a guide taxonomic tree as input (here the NCBI Taxonomy), the system recursively identifies core genes beginning from the most specific level (species or sub-species) at which completed genomes are available. Our algorithm determines the genes associated with terminal nodes (tree leaves, usually strains or sub-strains). We excluded 16S rRNA genes from this analysis as they are already well-established cores, and we likewise excluded tRNA genes because, despite high conservation, they typically possess very low phylogenetic information mainly because of their size (less than 100 nucleotides).

The algorithm first identifies core genes of the taxa occupying terminal nodes (i.e. organisms) by performing an ortholog search based on blastn hits [34]. Subsequently, ascending to higher nodes within the hierarchy, a blast nucleotide database is constructed using the gene sequences of all descendants and the genes of each descendant are searched against it. Genes with at least one homologous sequence in all taxa contained within the database are considered core genes and are removed (together with paralogs) from further computation to avoid placing the same gene in multiple core gene clusters. A minimum blastn word size of 10, e-value threshold of 0.001, and minimum alignment length of 75 were used in all analyses to maintain consistency with NCBI annotations for identifying genes in distant genomes. All orthologous (and paralogous) sequences are maintained in the set of core genes; the number of genes in each core gene set thus has a minimum cardinality equal to the number of direct descendant genomes. Using the core genes as representative of internal clades, the algorithm repeats this procedure for each clade in the tree (depth-first) as soon as core genes for all its direct descendants are available. This bottom-up process stops when no core genes are found for a clade or when the root is reached. The algorithm supports parallelism as independent clades can be analyzed concurrently.

### Genomic input data

We retrieved 1,236 prokaryotic annotate genomes as .ffn files from the NCBI ftp site. Genes contained only in plasmids were removed, genomes reported in multiple files (as different chromosomes) were concatenated, and each genome was identified using the NCBI Taxonomy. Apart from strictly parasitic species and obligate endosymbionts possessing as few as 200 genes, prokaryotic genomes typically carry at least 500 genes; genomes with fewer than 400 genes (8 in total) were thus excluded. This resulted in 1,107 taxa, for which the identifiers of all core genes are available for download at http://huttenhower.sph.harvard.edu/core-genes.

### Construction of microbial phylogenies

Sequence-based microbial phylogenies are usually derived using the universal 16S rRNA gene or a reduced set of concatenated genes present in a large fraction of the genomes [16]. Here we construct phylogenetic trees based on three strategies: using the standard 16S approach, using single core genes identified by the system described above, and using the functional information associated with most of the genes.

### Core genes phylogenetic trees

For each clade, we built a phylogenetic tree for each core gene. Only one paralog in each core gene set was used for this step; specifically, we select the paralog sequences with the highest similarity to the set of core genes without paralogs in the corresponding genomes. In this way, we consider the most conserved variant of each homolog; this procedure is not crucial, however, as preliminary testing showed that paralogous copies are almost always considerably more similar than orthologs in other clades, especially for high taxonomic levels. The orthologs representative of each taxa were aligned with MUSCLE 3.8.31 and a tree built from the alignments using the maximum-likelihood approach implemented in FastTree 2.1 [35]. The resulting trees are rooted using the midpoint criterion that maximizes the distance between the root and any leaf node (https://github.com/jhbadger/Newick-ruby) and are rendered using our module for circular cladogram visualization [36].

### 16S rRNA gene phylogenetic trees

The 1,107 16S rRNA genes for these organisms were analyzed using the pipeline above (MUSCLE for alignment, FastTree for tree building, and the midpoint approach for rooting the tree). The resulting phylogeny was also used to measure α-phylogenetic diversity by assessing the branch length of the highest node containing all taxa of the clade of interest using the PyCogent toolkit [37].

### Measuring distances between phylogenetic trees

In order to measure the similarity between phylogenetic trees, we constructed a distance matrix containing the shortest path branch length between each leaf (sequenced organism). This was transformed into a binary co-clustering matrix denoting as connected (i.e. belonging to the same phylogenetic sub-tree) the leaves with distances in the lowest 10^th^ percentile. This removes noise due to the variability of distances between nodes on very different sub-trees, as most meaningful information is included in the co-occurrences of nodes within small sub-trees rather than on the distances between very unrelated organisms. The distance between trees was finally computed as the Euclidean distance of the resulting binary adjacency matrixes. We used this measure of leaf co-clustering among trees in place of whole-tree metrics (like the Robinson-Foulds [38], [39]) to obtain a measure robust to internal branch lengths and branching criteria. These differ among the distinct algorithms we use for 16S, core genes, and functional phylogenies, thus making only the similarity of relative leaf position comparable among the three types of trees. We assessed the statistical significance of this similarity by comparing their distances to a set of baseline trees obtained by randomly shuffling leaf labels.

### Functional phylogenetic trees

To build phylogenetic trees representing the functional similarities of these 1,107 genomes, their shared protein families were summarized using COG families [27] as assigned by NCBI annotations. Each genome was summarized by an abundance vector of the number of genes in each COG family. Using these signatures, we built an abundance matrix with 4,685 columns (the total number of COGs) and 1,107 rows (the number of genomes). Columns summing to less than 10% of the number of genomes were removed, resulting in 3,514 columns (available at http://huttenhower.sph.harvard.edu/core-genes). We hierarchically clustered the matrix with average linkage and Manhattan distance using MeV and the functional trees are rooted and rendered as described for the 16S and core gene trees.

## Results

We integrated the sequence information of more than one thousand genomes to identify core genes conserved in increasingly large groups of microorganisms (clades), defining *core genes* as the set of orthologs ubiquitous within the genomes of a clade. The 16S rRNA gene, for example, is present in every bacterial organism (among others) and it is thus a core gene of the domain *Bacteria*. We functionally characterized the core genes and compared them to a functional phylogeny constructed by joining organisms with similar pathway and orthologous gene family complements. We finally compared phylogenies constructed using single families of core genes, using 16S rRNA sequences, and using the presence and absence of orthologous gene families.

### Core gene identification for 2,861 clades spanning 1,107 organisms

**Figure 1:**
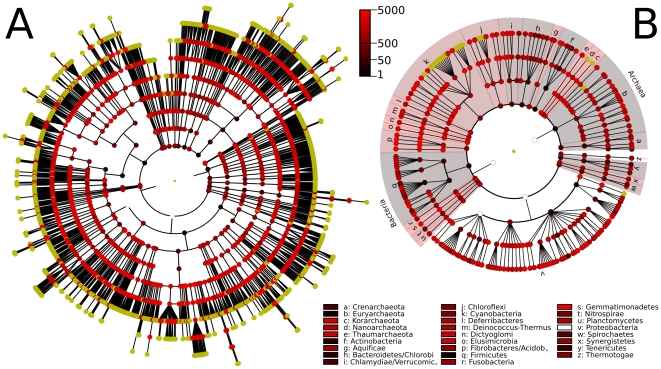
Evolutionarily conserved core gene sets calculated using the NCBI Taxonomy as a guide tree. In the circular cladograms each node represents a taxon, ranging from phyla (internal) to genera, species, and in some cases strains and sub-strains (external) with yellow leaf nodes representing taxa for which a complete genome is available. Core genes for higher-level clades are indicated by color (from black to red) proportional to the logarithm of the number of core genes, and white circles represent clades without cores. A) The full tree of core gene sets, representing 2,861 clades and 1,107 sequenced organisms. This tree includes the *Bacteria* and *Archaea* and results in cores that are functionally enriched for housekeeping genes including basic DNA and RNA operations. B) The core gene tree limited for visual clarity to the family. Note that our core gene discovery algorithm reflects for differences in phylogenetic depth and includes fewer core genes for broader clades spanning greater diversity.

We applied our method to all microbial genomes currently available from the NCBI, determining core genes conserved at each clade within the NCBI Taxonomy (Figure 1). Core genes cover hundreds of families and genera (e.g. *Streptococcus*, *Bacillus* and *Escherichia* for which more than 30 genomes are available) and include cores conserved up to the phylum level (one each for *Actinobateria*, *Firmicutes*, *Euryarchaeota*, 7 for *Bacteroidetes*, 61 for *Cyanobacteria*). The α-diversity (degree of sequence divergence, see Methods) of each clade is inversely proportional to the number of core genes (Spearman r = 0.94, p-value = 4e-16). The functional enrichments of core genes include housekeeping genes, but also genes related to posttranscriptional modifications, regulation, and uncharacterized genes (Table 1). The analysis of 1,107 organisms (leaves of the NCBI taxonomic tree) and 1,754 internal clades required 37 hours on an eight-core Intel i7 CPU (1.6GHz) using 2GB of memory. As described in Methods, our hierarchical procedure for identifying core genes and avoiding comparisons between all pairs of genes was crucial for achieving this degree of scalability.

**Table 1:** Overview of core gene set size and function for clades including at least 30 organisms.

Clade	Taxonomic level	α-diversity	# genomes	# core genes families	# S COGs	J K L COGsenrich. %		O COGsenrich. %	
Bacteroidetes/Chlorobi	group	5.2	48	3	0		33.3		38.6
Cyanobacteria	phylum	2.2	39	61	0		39.5	X	20.5
Proteobacteria	phylum	17.5	472	0	0		0.0		0.0
Firmicutes	phylum	10.8	233	1	0		100.0		0.0
Actinobacteria	phylum	5.9	110	1	0		0.0	X	100.0
Euryarchaeota	phylum	7.1	60	1	0		0.0		0.0
Bacteroidetes	phylum	4.5	37	7	0	X	55.8		28.7
Gammaproteobacteria	class	6.0	247	2	0	X	100.0		0.0
Actinobacteria	class	5.9	110	1	0		0.0	X	100.0
Clostridia	class	6.8	72	2	0	X	100.0		0.0
Alphaproteobacteria	class	5.4	109	0	0		0.0		0.0
Betaproteobacteria	class	2.3	48	70	0	X	55.6		8.2
Deltaproteobacteria	class	3.4	35	13	0	X	37.4	X	12.3
Epsilonproteobacteria	class	2.1	32	56	0	X	54.4		5.5
Bacilli	class	4.9	161	6	0	X	83.3		16.7
Actinobacteridae	subclass	5.0	102	2	0		32.7		67.3
Bacillales	order	2.8	79	28	0	X	45.4	X	14.2
Clostridiales	order	5.0	50	2	0	X	100.0		0.0
Lactobacillales	order	3.1	82	35	0	X	76.3		12.1
Actinomycetales	order	4.6	90	3	0	X	48.2		51.2
Rhizobiales	order	1.9	46	27	0	X	38.5		9.9
Pseudomonadales	order	1.0	31	159	0	X	44.5		6.8
Rickettsiales	order	1.9	30	16	0	X	53.6		0.0
Enterobacteriales	order	1.3	109	13	0	X	57.2		14.3
Streptococcaceae	family	1.8	52	316	11	X	46.1		5.9
Bacillaceae	family	1.9	42	217	3	X	33.4	X	7.2
Clostridiaceae	family	2.4	31	76	0	X	53.1	X	9.7
Enterobacteriaceae	family	1.3	109	10	0	X	56.8		14.4
Corynebacterineae	family	1.3	37	236	8	X	43.3		8.7
Streptococcus	genus	1.6	48	379	21	X	43.2		4.9
Bacillus	genus	1.5	32	334	6	X	28.9	X	6.5
Escherichia	genus	0.03	30	2192	23	X	17.8	X	4.7

We list for each clade, the α-diversity [37], the number of genomes, the number of core gene families, and the sizes of COG categories within these cores: S, unknown function; housekeeping, comprising J (translation, ribosomal structure, biogenesis), K (transcription), and L (replication, recombination, repair); and O, posttranslational modification, protein turnover, and chaperones. COG percentages refer to the fraction of orthologous core genes in the given functional category; statistically significant enrichments are computed using hypergeometric test with p<0.05.

### Number of core genes varies with phylogenetic diversity and genomic coverage

An overview of core genes for all taxonomic clades is shown in Figure 1; at least one core gene is discovered for each clade apart from *Archaea* and *Alphaproteobacteria* (and consequently *Proteobacteria* and *Bacteria*). The number of core genes is strongly determined by the phylogenetic α-diversity within each clade, in addition to a weak dependence on the number of genomes (see Table 1). Among the 4 phyla with the highest number of families, *Proteobacteria* spans the greatest diversity and possesses no core genes, whereas *Cyanobacteria, Firmicutes*, and the *Euryarchaeota* have 1, 61, and 1 core genes respectively. Note that the NCBI Taxonomy assigns sequenced genomes to different depths within its hierarchy (Figure 1A, mainly at the strain level but often at the sub-strain or genus level) and that our algorithm naturally accommodates this. Specifically, our computation adaptively starts at the lowest levels under which only leaf nodes are present, thus using any pre-existing taxonomy or phylogeny as a guide tree. The resulting core genes can then be used to reconstruct more refined sequence-based or functional phylogenies as described below.

### Functions of core genes include housekeeping and regulation

The functions of a clade's core gene set are a direct indication of the biological processes characteristic of that group of organisms, suggesting what functions may have been selected for to account for sequence-level conservation. We thus investigated the functional roles of core genes in order to characterize biological processes shared within each clade and maintained over the course of evolution. We compared the number of distinct COG functional categories present in a background of all microbial organisms to the core genes for each clade. We assigned a core gene family to a COG category if at least half of the orthologs were annotated to the category and assessed the resulting function assignments using standard hypergeometric enrichment.

The three most abundant COG categories (J, K, and L) represent the “information, storage and processing” group and implement general and indispensable functions including translation, ribosomal structure and biogenesis (J category), transcription (K category), and replication, recombination and repair (L category). These three COGs contained on average 22% of all genes in each organism, whereas they average almost half (46.2%) of the core genes for clades with more than 30 organisms (Table 1). This enrichment is itself an underestimate, since we did not include non-protein-coding genes (e.g. 16S and tRNAs) in these data, which belong to the J category and are known to form a substantial part of universal cores [16].

The majority of core genes have well-defined biological functions, often housekeeping roles in these COG J, K, and L categories, since these relatively ubiquitous genes have been studied extensively [40]. It is perhaps more surprising that many core genes are members of the O category, including posttranslational modification, protein turnover, and chaperones. The distribution of these genes is somewhat irregular, appearing relatively more often in clades with fewer core genes (statistical enrichment is achieved primarily for larger families due to reduced power in small sets of genomes). The orthologs of core genes in the O COG category show remarkable diversity and include chaperones, heme exporting proteins, hydrogenases, phosphatases and isomerases, confirming that the enrichment is category-wide and systematic.

Conversely, metabolism-related genes are underenriched in core genes relative to reference genomes, in agreement with the hypothesis that metabolic specialization within phylogenetic branches to adapt to varying environmental conditions is a major driver of evolutionary divergence [41]. It is also notable that several core gene sets include multiple genes of unknown function (S category). These occur somewhat surprisingly in some of the best-studied clades, including the *Streptococcus* and *Escherichia* genera; while these clades' cores are supported by an abundance of sequenced strain genomes, it is striking that many of the most strongly conserved genes among these organisms' pan-genomes are as yet uncharacterized. The uncharacterized genes include both proteins of completely undetermined function and also minimally characterized phosphokinases, stress response proteins, and DNA and RNA binding proteins, which might represent good targets for prioritization of future experimental follow-up.

### Functional phylogenetic analysis

Most comparative genomics focus on sequence-based phylogenetic methods using whole-genome, 16S rRNA gene, or core gene sequences as discussed above. Genetic similarity, however, is not always a precise proxy for phenotypic or functional similarity. Here we investigate the relationship between sequence-based and functional phylogenies, the latter constructed based on the presence and absence of protein function and pathways in organisms' genomes rather than on the sequences of the genes encoding these functions. Since this functional similarity is based on the presence or absence of protein functions rather than on strongly conserved, sequence-similar gene families, we adopted the broad COG [27] database as a descriptor for orthologous protein clusters. In summary, we find that the co-presence or absence of specific functions often, but not always, correlates with evolutionary relationships, as observed above in the functional enrichments of core gene sets. Functional phylogeny thus provides a complementary perspective that highlights relationships between divergent organisms potentially occupying similar environmental niches.

**Figure 2:**
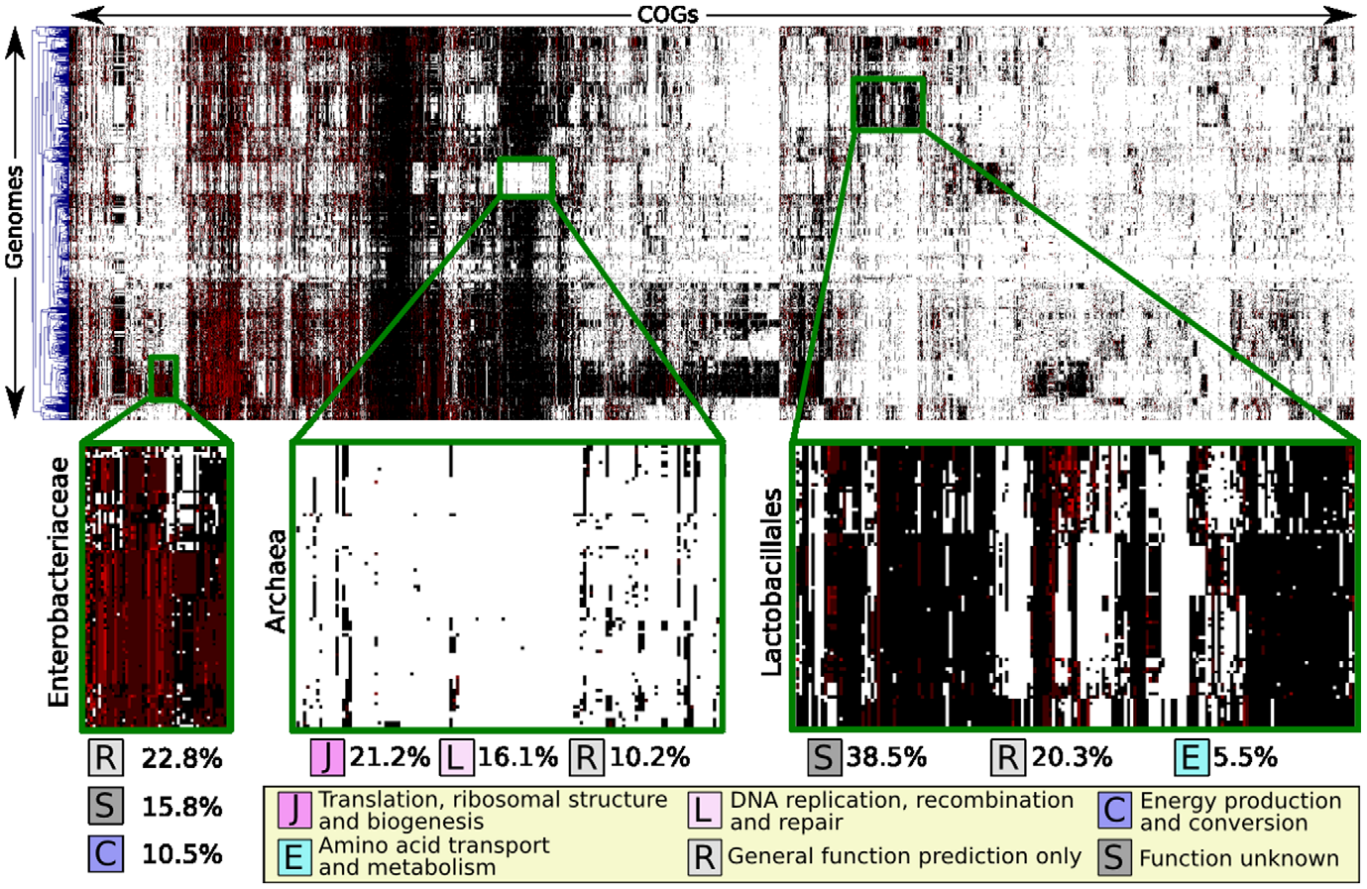
Hierarchical clustering of organisms based on the COG gene families present in their genomes. Heat maps represent individual COG orthologous gene family abundances (columns) for each taxon (rows); absent gene families are white, single copy families are black, and multicopy families red. The resulting functional phylogeny represents a type of phylogenetic profiling and clearly highlights functional characteristics specific to groups of organisms independent of their evolutionary relatedness. In many cases, evolutionary and functional similarities are highly correlated; three representative clusters are enlarged in green boxes and the three most abundant COGs for each cluster are reported. Note that the absence of the bacterial ribosome and DNA maintenance machinery from the *Archaea* is readily apparent from such data, and that the *Enterobacteriaceae* and *Lactobacillales* both include striking large clusters of strongly conserved uncharacterized genes.

**Figure 3:**
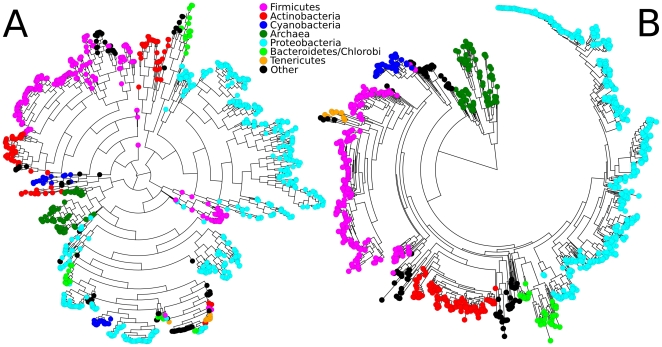
Contrasting a functional phylogeny built using shared gene families with a 16S gene sequence phylogeny. 1,107 sequenced microbes clustered using (A) the cooccurence of COG orthologous gene families (see Figure 2) or (B) 16S gene sequence similarity (using Muscle and FastTree [35]). Phyla from the NCBI Taxonomy are indicated by color. While overall organismal similarities are maintained at both the functional and sequence levels, the two phylogenies provide distinct perspectives on organismal relatedness. Some clades like the *Archaea* and *Firmicutes* form very distinct sub-trees, whereas others like *Bacteroidetes/Chlorobi*, *Actinobacteria* and *Cyanobacteria* show high 16S similarity relative to their functional similarity.

Figure 2 reports the functional phylogeny formed by hierarchically clustering (using average linkage with Manhattan distance) 1,107 genomes based on the abundance (or absence) of each of the 3,514 COG orthologous gene families with total abundance equal to at least 10% of the number of available genomes. As indicated by previous phylogenetic profiling studies [38], [39], the resulting clusters of genomes possessing similar COG gene families often correspond to phylogenetically related organisms. These include the cases highlighted in Figure 2 comprising metabolic and transport pathways in the *Enterobacteriaceae* and *Lactobacillales*; conversely, many clades lack COGs abundant in other genomes, exemplified by the absence of bacterial ribosomal and DNA maintenance machinery in the *Archaea*. It is striking that, in agreement with the core gene sets found above, several of these functionally discriminative modules are made up largely of uncharacterized genes (as highlighted for the *Lactobacillales*). Also key to functional phylogenetic analyses is that this measure of organismal similarity does not always mirror sequence similarity, as is for example the case with the *Bacteroides* and *Chlorobi* phyla. The 16S sequences of these two phyla are very similar (see Figure 3B) and support their taxonomic union, but the difference in the functional modules they encode is comparable to the differences between other phyla.

With this hierarchical clustering based on COG profiles, we reconstructed a microbial functional phylogenetic tree and compared it to a 16S rRNA-based phylogeny (Figure 3) as commonly used for comparative genomics [25], [35], [42]. Most clades are well-defined in both phylogenies, although the two trees show interesting differences both in the relative depth and in the internal consistency of several clades. In particular, order-level clades functionally cluster together more consistently than do phyla; this suggests that, even for phyla with ancient divergence indicated by 16S sequence dissimilarity, more recent adaptation to common environments is quite visible in these functional data at approximately the order level. *Firmicutes* and *Proteobacteria*, for example, are tightly clustered in the 16S phylogeny, but the two phyla encompass substantially greater functional diversity than sequence divergence. Similarly, several clades such as the *Actinobacteria* and *Cyanobacteria* are so functionally divergent that they fall into distinct subgroupings within other phyla. This functional perspective thus allows phenotypic features to be rapidly observed in large collections of microorganisms. Unsurprisingly, the only clade that shows consistent functional and sequence isolation is the Archaea, a domain well known to be both functionally and genotypically distinct from bacteria [43], [44].

### Relating functional and sequence phylogenies

A broad case in which functional profiling tended to differ from phylogenetic relationships was within clades with many closely related, low-diversity genomes. This is the case, for example, in the *Proteobacteria* (Figure S2), in which many strains are near-indistinguishable at the 16S sequence level, but carry substantial differences in genomic functional potential (as indicated by branch length in the functional phylogeny). This phenomenon is present qualitatively in each of these examples (e.g. among the Firmicutes in Figure 3) and may represent a degree of recent, ongoing horizontal gene transfer among clades with putative “open” pan-genomes [45], [46].

To quantitatively analyze the relation between functional, 16S rRNA, and, in the next section, core gene phylogenies, we considered three well-studied clades in the Enterobacteriaceae family: Escherichia, Shigella, and Salmonella. At the sequence level, Escherichia and Shigella are a common example of clades undistinguishable by 16S, and this is itself strong evidence supporting their common origin and phylogenetic co-grouping [47], [48]. From a functional perspective, however, the two clades possess substantial diversity, and the resulting phenotypic differences have been clinically well characterized. Specifically, it has been proposed that groups of Shigella organisms derived from independent Escherichia strains by means of repeated, similar horizontal transfer events [49]. This is thus one example of functional divergence occurring much faster than nucleotide sequence divergence, thereby ensuring that functional and sequence phylogenies will yield distinct information for the two clades.

The opposite phenomenon also occurs during convergent evolution when phylogenetically distant genomes show an unexpectedly similar functional potential. One such example is the group of “minimal” clades (genomic sizes smaller than 1 Mb for most strains) comprising the Borrelia, Mycoplasma, Chlamydia, and Buchnera genera. Their sequence dissimilarity (Table S1) is comparable to the distance between different orders or phyla, whereas their functional potential suggests a much tighter relation ad detailed in Table S1 and in the next paragraph. Along with their small genomic size, this agrees with their evolution towards a minimal genome from less related species by means of non-essential gene loss and, for obligate parasites, partial core genome depletion [50], [51]. It is thus reasonable to hypothesize that these genera functionally converged from distant non-minimal genomes as a consequence of similar environmental pressures, and this biological hypothesis can be directly generated by the automated comparison of functional and sequence phylogenies.

Such observations based on the uncoupling of functional and sequence phylogenies are typefied by the two microbial trees of Figure 3. Specifically, after normalizing the two trees with respect to total branch length, we summarized the trees as all-versus-all organismal dissimilarity matrixes of shortest path branch length. Averaging the resulting distances between genomes in pairs of genera, we obtained an overall functional distance of 9.84±0.53 between Escherichia and Shigella, which is much larger than their inter-genus 16S distance of 0.14±0.06 as well as their intra-genus 16S distances of 0.12±0.06 for Escherichia and 0.16±0.07 for Shigella. Other genera also show this behavior, including the Rhodopseudomonas and Nitrobacter with 0.63±0.18 and 24.7±0.16 average 16S rRNA and functional distance, respectively, and the Staphylococcus and Bacillus genera with 16S and functional divergences of 2.38±0.48 and 27.7±3.8. In contrast, as discussed above, the “minimal” organisms are instead much more phylogenetically distant (minimum 16S rRNA average distances of 15.9±2.2 between Borrelia and Chlamydia) than functionally diverse (maximum functional average distances of 2.6±0.2 between Mycoplasma and Buchnera; see Table S1 for complete distance matrices). These contrasts provide a rapid, automated computation by which putative convergent and recent divergent evolution can be detected.

### Comparing 16S and core gene phylogenies

**Figure 4:**
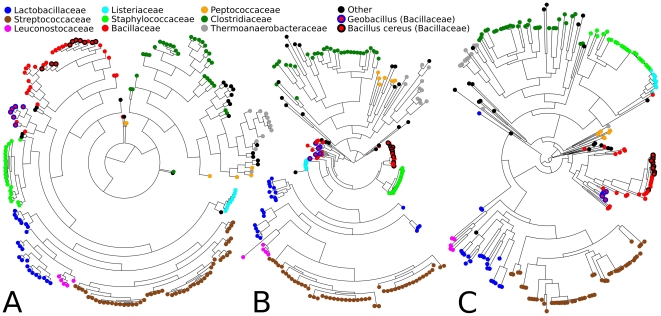
*Firmicutes* phylogenies obtained using functional clustering, 16S rRNA gene sequence, or core gene sequence. Colored leaves represent taxonomic orders. Trees generated using (A) functional similarity of COGs as detailed in Figure 2, (B) 16S rRNA gene similarity, and (C) core gene sequence similarity (for excinuclease ABC subunit A, the only core gene found for *Firmicutes*). Note that while overall tree structure is comparable for the three methods, functional phylogeny and the excinuclease ABC subunit A core gene sequence correctly assign organize the *Bacillus cereus* group with other *Bacillus* and *Geobacillus* genera within the *Bacillaceae*.

The 16S rRNA is only one gene that fits the requirements for a consistent phylogenetic marker, and the sequences of one or more core genes (as derived above) can also be used to derive putative evolutionary relationships [3], [7]. As performed using manually curated cores in [7], we compared phylogenetic trees obtained with full-length 16S gene sequences with the core gene families found by our method. Here, in contrast to recent approaches, we evaluate the phylogenetic information contained in clade-specific single core gene families, rather than concatenating the sequences of several manually curated conserved genes; we also use automatically identified clade-specific core genes so as to avoid relying on a small set of universal proteins. We performed specific comparisons for the *Firmicutes* (using excinuclease ABC subunit A as a core gene, Figure 4), for the *Actinobacteria* (using Chaperonin GroEL, Figure S1 in additional material) and for *Gammaproteobacteria* (using chorismate synthase and DEAD/DEAH box helicase domain protein as two different cores, Figure S2). Measuring distances between trees as reported in Methods, the two sequence-based phylogenies and the function-based approach agreed significantly in their grouping of taxa (average distances of 35±2 for *Actinobacteria*, 54±14 for *Gammaproteobacteria* and 48±3 for *Firmicutes*, relative to random baselines of 44±1, 101±2 and 94±1), all with p-values <0.001 (based on 1,000 randomizations). For all three clades, the three approaches demonstrated surprising consistency in both the global and local structure of the resulting phylogenetic trees, particularly between the core gene and functional approaches.

In addition to this overall consistency, we further found several cases in which core genes provided additional information beyond the resolution of 16S phylogenies. For *Firmicutes*, core genes (as well as functional phylogenies) correctly placed the *Bacillus cereus* group in the same subtree with other *Bacillus* genera and with *Geobacillus* (particularly the core gene approach), whereas a 16S phylogeny intercalates their sub-trees with other clades (Figure 4). A global perspective on these differences is shown in Figure S2, which suggests that the excinuclease core gene specifically possesses more power than 16S for distinguishing highly similar organisms than 16S (see points above the bisector line in Figure S3), in part due to the resolution offered by the length of the excinuclease gene (approximately 3kb, double that of 16S). Notable differences are also evident at the family level (Figure 4), in which the Bacillaceae are placed much closer to the Staphylococcaceae and Listeriaceae in the 16S phylogeny. Within the Enterobacteriaceae, we quantitated these differences for the Escherichia and Salmonella species, building clade-specific phylogenetic trees using the 16S rRNA and the 10 core genes found for this clade (Table 1). After normalizing with respect to total branch length in each tree, we compared the distances between Escherichia and Salmonella genomes with the intra-clade distances for the two species. The ratio between average inter- and intra-species distances for 16S is 5.0, whereas nine of the ten core genes have higher values with the four most striking cases being the 30S ribosomal protein S7 gene (ratio 34.2), the CTP synthetase gene (ratio 28.7), the preprotein translocase subunit SecA gene (ratio 18.0), and the ATP-dependent protease ATP-binding subunit ClpX gene (ratio 16.5). All of these automatically derived core genes thus have greater resolution for accurately identifying phylogenetic relationships within the family, including among the Escherichia themselves.

Taken together with the data above, this demonstrates the complementary functional and evolutionary information that can be conveyed by contrasting single-marker phylogeny (e.g. from 16S sequences) with functional phylogenies and with the conserved core genes derived in high-throughput.

## Discussion

We describe here a computational approach for identifying strongly conserved core genes for microbial clades constructed efficiently from a large collection of sequenced genomes. 2,861 core gene sets covering 1,107 genomes were constructed recursively for clades at increasingly broad levels beginning with a user-defined taxonomy. Cores resulting from this process were used to refine clade-specific phylogenies based on standard sequence similarity. The number of core genes per clade was, as expected, inversely correlated with phylogenetic diversity, and *Archaea* and *Alphaproteobacteria* were the only taxa which no core genes were found. This lack of strongly conserved genes at high phylogenetic levels is not surprising, as such a completely universal protein core is best identified by local protein similarity, whereas our focus here is on medium-to-highly related clades using nucleotide similarity. At the genus, species, or strain level, this highlights the importance of focusing on less-universal genes whose diversity is more indicative of local, rather than global, phylogenetic relationships. The functional composition of these core gene sets further provides an informative descriptor of the biological processes strongly conserved within each clade, often consisting of housekeeping genes but also including a variety of regulatory proteins, chaperones, and uncharacterized genes. Finally, while molecular phylogenies constructed using sequence similarity of either core genes or the standard 16S rDNA marker were comparable, they are complemented by functional phylogenies constructed using the presence or absence of orthologous gene families within microbial genomes.

While phylogenetic profiling has become a mainstay of comparative genomics, grouping together genes that are co-conserved over evolutionary time [28], [29], [30], [31], there has been relatively little work on the “perpendicular” approach of grouping organisms based on the co-occurrence of protein functions or pathways [32], [33], [52], in part because its usefulness was unclear prior to the current availability of thousands of microbial genomes [53]. Functional phylogenies using this approach provide an interesting alternative to molecular phylogenies for exploring large groups of microbial genomes. Since the behavior and, in particular, pathogenicity of microbial organisms depends on the functional modules they possess, the comparison between functional and phylogenetic distances can provide specific insights into the relationships between distantly related microorganisms occupying similar ecological niches, pathogens and their nonvirulent counterparts, and for investigating the functions implemented by entire microbial clades or communities. In particular, the results illustrated here show that uncoupling between functional and phylogenetic similarities can characterize functionality within highly related clades such as the Escherichia/Shigella group or, conversely, among the phylogenetically diverse “minimal” organisms. In the former case, this also shows that core gene phylogenies can in some cases be more accurate than 16S and universal marker phylogenies, since clade-specific core genes are likely to possess a less diverged and diffuse phylogenetic signal within closely related clades.

Functional phylogeny groups together organisms with similar complements of orthologous protein families or functional modules; alternatively, conserved microbial function can be studied by characterizing the functional enrichments of core genes ubiquitous within clades related by sequence phylogeny [15]. Here, particularly for clades with substantially many sequenced genomes, such core genes are enriched for housekeeping functions as expected [54], and also for posttranslational modification, protein turnover, chaperones, and many uncharacterized gene families. The functional characterization of proteins shared within a clade provides an interesting bridge between insight into the group's ancestral functions [55], [56] and the functional selection imposed by environments its members currently inhabit [57]. Moreover, the same functional profiling approach can be used to investigate the minimal set of genes necessary for microorganismal survival, to characterize the core functional categories needed for a microbial community [58] to survive in diverse or particular environments [59], or for a clade to behave pathogenically within the human body.

By systematically and efficiently determining conserved families of open reading frames, the computational system proposed here for core gene discovery is scalable with respect the number of input genomes (currently more than 1,100) and adaptable with respect to the initial microbial input taxonomy. Hierarchical analysis of organisms accordingly to an approximate initial taxonomic classification not only allowed us to identify core genes throughout all levels of the microbial tree of life, but was also crucial in dramatically lowering the number of sequence similarity searches required for detecting core gene families. The method is thus capable of processing the increasing catalog of sequenced microorganisms and of applying subsequent functional and phylogenetic analysis pipelines to the resulting refined sets of core genes. The initial set of all 2,861 core gene families is made available for download within our supplement, and future work will include the development of a web interface for exploring this large dataset interactively (core genes, functional profiles, and phylogenetic trees) and application of the system to microorganismal quantification within metagenomic data.

## Supporting Information

Figure S1 **Phylogenies for the phylum Actinobacteria obtained using functional clustering, 16S rRNA gene sequence, and core gene sequences.** Colored leaves represent taxonomic orders. Trees generated using (A) functional similarity of COGs as detailed in Figure 4, (B) 16S rRNA gene similarity, and (C) core gene sequence similarity for chaperonin GroEL, the only core gene found among all Actinobacteria.
(TIFF)

Figure S2 **Phylogenies for the phylum Gammaproteobacteria obtained using functional clustering, 16S rRNA gene sequence, and core gene sequence.** Colored leaves represent taxonomic orders. Trees generated using (A) functional similarity of COGs as detailed in Figure 4, (B) 16S rRNA gene similarity, (C) core gene sequence similarity (for chorismate synthase, one of the two core genes found for Gammaproteobacteria), and (D) core gene sequence similarity for DEAD/DEAH box helicase domain protein, the second of the two core genes found for Gammaproteobacteria.
(TIFF)

Figure S3 **Comparison between the phylogenetic distances of organisms in the phylum Firmicutes computed using the 16S rDNA and the excinuclease ABC subunit A core gene.** Distances among all Firmicutes bacteria were computed using phylogenetic trees constructed from each of these two genes, with branch lengths normalized to total length one. The contrast highlights the higher resolution of the excinuclease core gene for very related organisms, as these occur with 16S distances uniformly smaller than 0.1 but spanning a core gene range up to 0.4.
(TIFF)

Table S1 Average functional and sequence distances between four minimal genera. The reported values are computed on the corresponding normalized phylogenetic trees, averaging the distances of pairs of organisms spanning different genera (off-diagonal) or within the same genus (diagonal).
(XLSX)
