# Supplementary figures and images for: Toward an Efficient Method of Identifying Core Genes for Evolutionary and Functional Microbial Phylogenies
Source: PLoS One. 2011 Sep 12;6(9):e24704. doi: 10.1371/journal.pone.0024704 (PMC3171473; doi:10.1371/journal.pone.0024704)

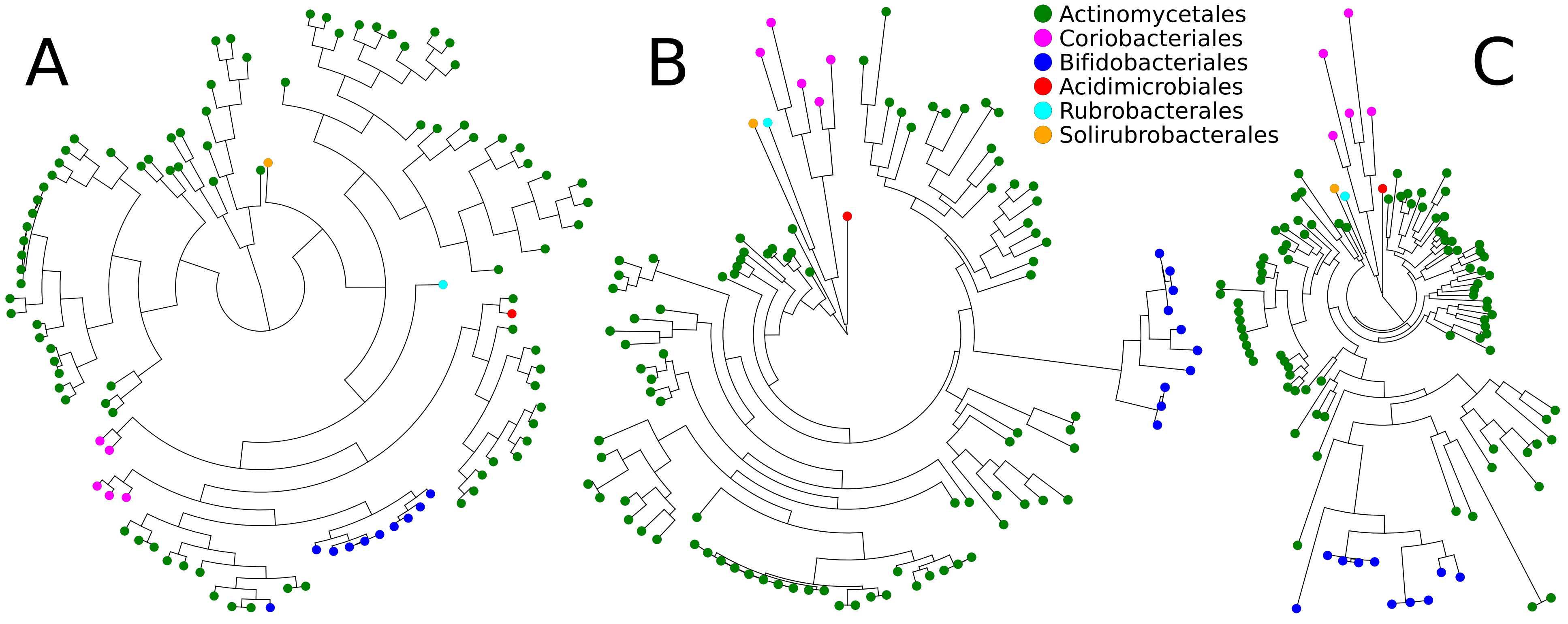

Supplement: Figure S1 — Phylogenies for the phylum Actinobacteria obtained using functional clustering, 16S rRNA gene sequence, and core gene sequences. Colored leaves represent taxonomic orders. Trees generated using (A) functional similarity of COGs as detailed in Figure 4, (B) 16S rRNA gene similarity, and (C) core gene sequence similarity for chaperonin GroEL, the only core gene found among all Actinobacteria. (TIFF) [file pone.0024704.s001.tiff]

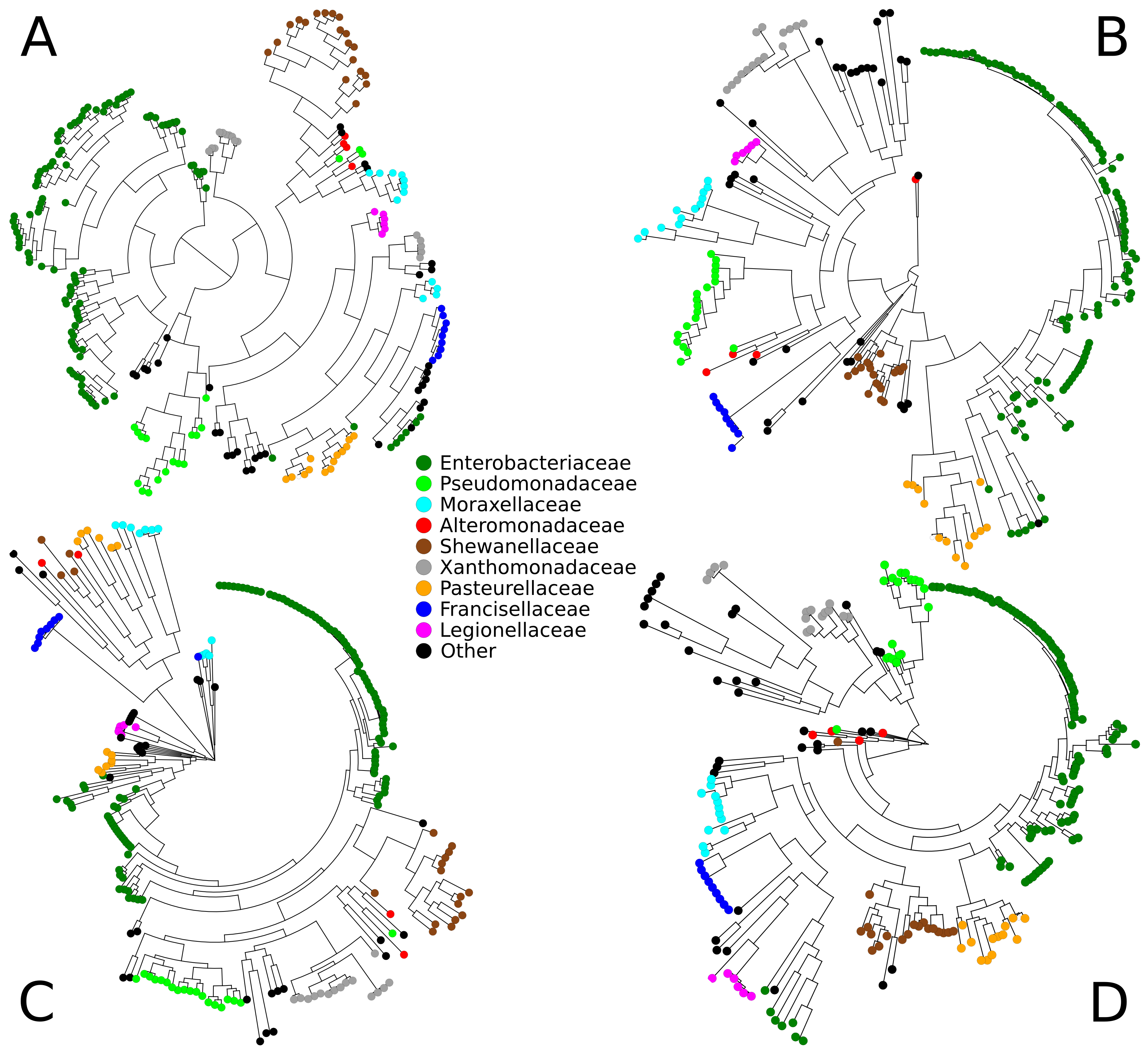

Supplement: Figure S2 — Phylogenies for the phylum Gammaproteobacteria obtained using functional clustering, 16S rRNA gene sequence, and core gene sequence. Colored leaves represent taxonomic orders. Trees generated using (A) functional similarity of COGs as detailed in Figure 4, (B) 16S rRNA gene similarity, (C) core gene sequence similarity (for chorismate synthase, one of the two core genes found for Gammaproteobacteria), and (D) core gene sequence similarity for DEAD/DEAH box helicase domain protein, the second of the two core genes found for Gammaproteobacteria. (TIFF) [file pone.0024704.s002.tiff]

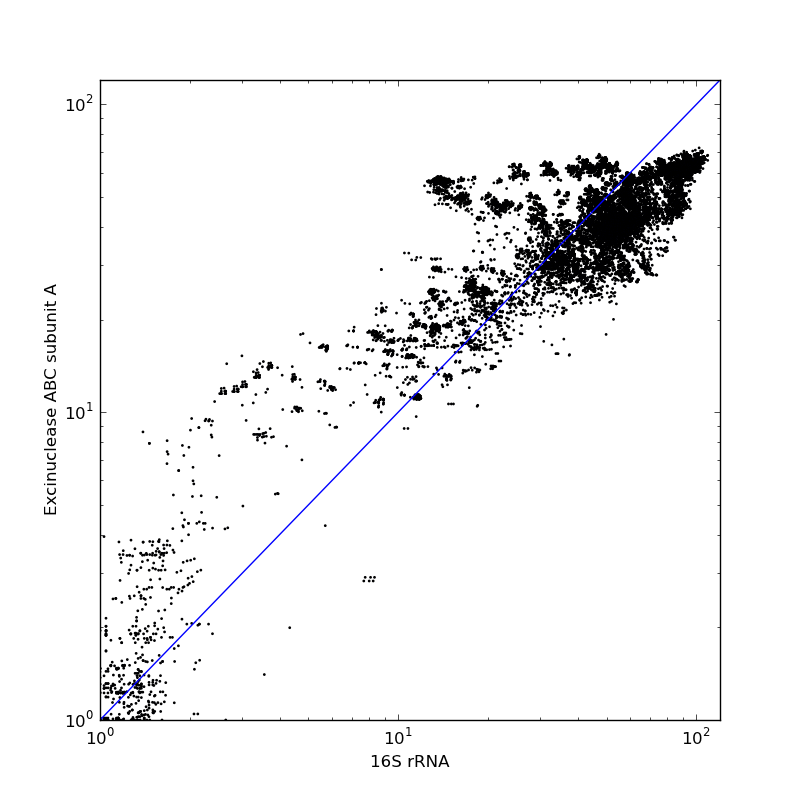

Supplement: Figure S3 — Comparison between the phylogenetic distances of organisms in the phylum Firmicutes computed using the 16S rDNA and the excinuclease ABC subunit A core gene. Distances among all Firmicutes bacteria were computed using phylogenetic trees constructed from each of these two genes, with branch lengths normalized to total length one. The contrast highlights the higher resolution of the excinuclease core gene for very related organisms, as these occur with 16S distances uniformly smaller than 0.1 but spanning a core gene range up to 0.4. (TIFF) [file pone.0024704.s003.tiff]
